# Plasma proteomics analysis of Chinese HIV-1 infected individuals focusing on the immune and inflammatory factors afford insight into the viral control mechanism

**DOI:** 10.3389/fimmu.2024.1378048

**Published:** 2024-05-10

**Authors:** Wanqi Ni, Li Ren, Lingjie Liao, Dan Li, Zhenwu Luo, Meiling Zhu, Ying Liu, Hui Xing, Zheng Wang, Yiming Shao

**Affiliations:** ^1^ National Key Laboratory of Intelligent Tracking and Forecasting for Infectious Diseases, National Center for AIDS/STD Control and Prevention, Chinese Center for Disease Control and Prevention, Beijing, China; ^2^ Autoimmune Department, BioRay Pharmaceutical Co., Ltd., San Diego, CA, United States; ^3^ Changping Laboratory, Beijing, China

**Keywords:** HIV, long-term non-progressor (LTNP), plasma, proteomics, immune balance regulation, cytokine, neutralizing activity

## Abstract

**Background:**

Long-term non-progressors (LTNPs) with HIV infection can naturally control viral replication for up to a decade without antiretroviral therapy (ART), but the underlying mechanisms of this phenomenon remain elusive.

**Methods:**

To investigate the relevant immune and inflammatory factors associated with this natural control mechanism, we collected plasma samples from 16 LTNPs, 14 untreated viral progressors (VPs), 17 successfully ART-treated patients (TPs), and 16 healthy controls (HCs). The OLINK immune response panel and inflammation panel were employed to detect critical proteins, and the plasma neutralizing activity against a global panel of pseudoviruses was assessed using TZM-bl cells.

**Results:**

The combination of IL17C, IL18, DDX58, and NF2 contributed to discriminating LTNPs and VPs. IL18 and CCL25 were positively associated with CD4^+^ T cell counts but negatively correlated with viral load. Furthermore, CXCL9 and CXCL10 emerged as potential supplementary diagnostic markers for assessing the efficacy of antiretroviral therapy (ART). Finally, TNFRSF9 displayed positive correlations with neutralization breadth and Geometry Median Titer (GMT) despite the lack of significant differences between LTNPs and VPs.

**Conclusion:**

In summary, this study identified a set of biomarkers in HIV-infected individuals at different disease stages. These markers constitute a potential network for immune balance regulation in HIV infection, which is related to the long-term control of HIV by LTNPs. It provides important clues for further exploring the immune regulatory mechanism of HIV.

## 1 Introduction

Since its discovery in the 1980s, acquired immunodeficiency syndrome (AIDS), which is caused by the human immunodeficiency virus (HIV), has continued to cause severe threats to worldwide public health (1, 2). At present, there are approximately 39 million individuals globally living with HIV, with roughly 30 million of them currently undergoing antiretroviral therapy (ART) (3). While ART effectively controls viral replication and keeps plasma viral load (VL) below detection limits, the persistence of the HIV reservoir requires patients to remain on lifelong medication (4, 5). Without ART, most individuals may experience viral viremia, leading to severe immune dysfunction due to sustained HIV replication (6). Especially, long-term non-progressors (LTNPs) can spontaneously maintain a relatively stable state of health for decades without ART (7), and are characterized by consistently high CD4^+^ T-cell counts (>500 cells/mm^3^), low viral loads, rare HIV-associated symptoms, and robust immune responses (8). Therefore, research on LTNP can enhance our comprehension of the immune control mechanisms underlying HIV infection and enable the identification of potential biomarkers for HIV treatment and vaccine development.

Specific immunogenetic factors in LTNPs may contribute to the immunological clearance of HIV. LTNPs frequently have HLA B57*, B58* and B27* alleles, and a combination of HLA-B57* and IFNL4 polymorphisms is associated with LTNPs (9). IL-18 and IP-10 plasma levels with activated CD8^+^ cells have also been proposed as potential LTNP indicators (10). Besides, neutralizing antibody, CCR5-Δ32bp genotype and other host restriction factors such as APOBEC3G, Tetherin and Trim5α also contribute to the viral control (11, 12). Although certain immunological aspects of LTNPs have been found, some concerns remain unresolved. Can LTNPs sustain immune homeostasis comparable to ART-treated patients (TPs)? Is it possible to uncover new biomarkers associated with disease stages in Chinese LTNPs and viral progressors (VPs)? The search for immune differences between typical VPs, LTNPs and TPs could provide relevant knowledge and identify new targets for predicting disease prognosis.

A certain level of inflammation is also present in LTNPs due to the inability of virus eradication, despite their robust immune system maintaining immune function (13). PD-L1, VEGFA, LAP TGF β-1 and TNFRSF9 have been identified as potential predictors of inflammation levels in HIV-infected individuals undergoing ART (14). CXCL11, CXCL9, TNF, CXCL10, and IL18 are associated with abnormal T-cell regulation during chronic HIV-1 infection (15). Nevertheless, these reports mainly focused on inflammation levels post-ART and their relationships with clinical stages. There is a lack of comprehensive research on the expression levels across different populations, including LTNPs, VPs, TPs and HCs.

In this research, we intend to evaluate the immune and inflammatory factor levels using proximity extension assays (PEA) technology in the plasma of LTNPs, VPs and TPs. By combining clinical indicators such as plasma neutralizing activity, CD4^+^ T-cell counts and viral load, this research aims to gain insights into the immune regulation mechanisms against HIV and to identify specific biomarkers associated with disease progression and ART efficacy.

## 2 Methods

### 2.1 Study subjects

Plasma was collected separately from HIV-1-infected individuals from the established cohort in Henan Province, Anhui Province, and Zhejiang Province. LTNPs and VPs were infected by HIV-1 clade B through unregulated commercial blood donation in the middle of the 1990s in Henan or Anhui Province, and the plasma was collected in 2007 or 2008. LTNPs and VPs never received ART until 2008. LTNPs had CD4^+^ T-Cell counts over 500 cells/μL and viral load below 10^4.5^ copies/mL, while VPs had lower CD4^+^ T-Cell counts (<400 cells/μL) and higher viral load (10^3.4-5.7^ copies/mL). TPs were infected by B, CRF01_AE or CRF07_BC through MSM (Men who have sex with men) or HST (Heterosexual transmission) in Henan Province, Anhui Province and Zhejiang Province in 2013-2019, and the plasma was sampled in 2-5 years after ART (AZT/D4T+3TC+EFV) initiation. Besides, the plasma of HIV-negative people was used as negative control in the plasma proteomic assay.

### 2.2 Olink proteomic assay

In this study, the Proximity extension assays (PEA) technology of Olink Proteomics AB immune response panel (Cat:95320 Olink Bioscience AB, Uppsala, Sweden) and inflammation panel (Cat:95302 Olink Bioscience AB, Uppsala, Sweden) were used to measure the concentrations of 184 plasma cytokines in all individuals including 92 immune cytokines and 92 inflammatory cytokines. Protein arrays were performed on plasma samples, in which 1% TritonX-100 was added for inactivation for two hours. Then, plasma samples were treated according to the manufacturer’s instructions and were loaded into the primed 96×96 Dynamic Array IFC. Following that, chips were dealt with the Olink Protein Expression 96×96 Program in the Fluidigm Biomark TM Reader according to the manufacturer’s instructions. The protein analysis was reported as normalized protein expression levels (NPX), which were Ct values normalized by subtracting values for extension and inter-plate control. The scale was shifted using a correction factor (normal background noise) and reported in the Log2 scale. The NPX values of each sample were deposited in the **Supplementary Materials** (**Datasheet 1**). The sample that did not pass the OLINK quality control was excluded in subsequent analyses.

### 2.3 Bioinformatics analysis

After background correction and normalization of NPX, we utilized the ‘limma’ package (16), based on the Bayesian statistical method, to analyze the differential expression between two groups of individuals. Proteins with p-value < 0.05 and |Fold Change| > 1.5 were defined as differentially expressed proteins (DEPs). Each DEP is colored on the volcano plots of all the detected proteins. The DEPs screened in the difference analysis were subjected to Kyoto Encyclopedia of Genes and Genomes (KEGG) pathway enrichment analysis and Gene Ontology (GO) enrichment analysis based on the ‘clusterprofiler’ package (17), and the enrichment results with p-value < 0.05 and the top ten enriched pathways were visualized. The online tool STRING version was used to analyze and predict interactions between DEPs and visualized by CytoScape version 3.9.1 (18, 19).

### 2.4 Neutralization assay of plasma

The neutralizing activity of the plasma against 12 global panel viruses, including clades A, B, C, G, CRF07_BC and AC, was assessed using a conventional TZM-bl cell-based and Env-pseudotyped neutralization test (20, 21). In brief, plasma samples was three-fold serially diluted with Dulbecco’s modified Eagle’s medium (DMEM; Cat.10-013-CV, HyClone, USA). Serially diluted plasma (50 μL) and 50μL of pseudovirus (200 TCID_50_) were mixed and placed in the wells of a 96-well plate. The plate was then incubated in a cell incubator for one hour. The viral controls (TZM-bl cells and the pseudovirus) and cell controls (TZM-bl cells only) were set concurrently. Then, 100 μL of TZM-bl cells (10^4^) in DMEM containing 25 μg/mL DEAE-dextran (Cat. D9885, Sigma, USA) were added to each well. The plates were incubated in a 5% humidified solution at 37°C for 48 hours. After that, 150 μL of the supernatant was removed from each well, and 100 μL of the Bright-Glo luciferase reagent (Cat. 6066769, Promega, USA) was added into each well subsequently. Then, 150 μL of the cell lysate was transferred to a black plate after 2.5 minutes of incubation, and a Victor 3 luminometer (PerkinElmer, USA) was used to measure luminescence. The plasma reciprocal dilution that reduced the infection by 50% was calculated as the half-neutralization titer (NT_50_). The neutralization breadth was determined by the number of neutralized viruses (NT_50_ > 20) ×100%/12 viruses. NT_50_ value < 20 (lowest sample dilution tested) was assigned a value of 10 for GMTs calculation. Geometry Median Titer (GMT) is derived from the average NT_50_ of 12 viruses (21).

### 2.5 Statistical analysis and visualization

The data were analyzed using R software version 4.2.3 and GraphPad Prism version 9.5.0 (GraphPad Software, Inc., CA, USA). Student’s t-test was employed to compare differences between two groups, while two-way analysis of variance was utilized to analyze differences among three or more groups. Pearson or Spearman correlation analysis assessed the correlation between two variables. Wilson/Brown’s method was used for receiver operating characteristic curve (ROC) analysis. A significance level of p-value < 0.05 was considered statistically significant (two-tailed). GraphPad Prism version 9.5.0 is used to plot violin plots, ROC curves, and box plots. Moreover, the following R packages are used to visualize the results of the bioinformatics analysis: the ‘VennDiagram’ package for the Venn plot (22), and ‘ggplot2’ for others (23).

## 3 Results

### 3.1 Characteristics of participants and quality control of NPX data

Briefly, 16 LTNPs and 14 VPs without ART, 17 TPs, and 16 HCs were selected based on viral load, CD4^+^ T-cell counts, and treatment status. The characteristics of enrolled individuals are provided in **Table 1**. The median ages of LTNPs, VPs, TPs and HCs are 47 years, 44 years, 49 years and 29 years, respectively. The median CD4^+^ T-cell counts of LTNPs, VPs and TPs are 571 cells/mm^3^, 291 cells/mm^3^ and 601 cells/mm^3^, respectively. Median viral loads of LTNPs and TPs are lower than detectable levels (LDL), while VPs present median viral loads of 10^4.54^ copies/mL. After OLINK’s quality control, 13 LTNPs, 11 VPs, 16 TPs and 15 HCs in the immune response panel, and 16 LTNPs, 14 VPs, 16 TPs and 15 HCs in the inflammation panel passed. Further analysis was based on the qualified data.

**Table 1:** Characterization of enrolled subjects in this research.

Characteristics	Long Term Non-Progressors	Viral Progressors	Treated Patients	Healthy Controllers	p-value
n	16	14	17	16	
Age, median (IQR)	47 (42-50)	44 (38-48)	49 (36-63)	29 (24-32)	<0.0001^a^
Male (%)	5 (31)	4 (29)	12 (71)	5 (31)	0.0405^b^
HIV-1 subtype n, (%)	B	B	B (10/17) 01AE (1/17) 07BC (2/17) unknown (4/17)	NA	0.0244^b^
CD4^+^ T-Cell Counts (cells/mm^3^), median (IQR)	571(518-718)	291(192-325)	601(462-692)	NA	<0.0001^c^
Viral Load (log_10_copies/mL), median (IQR)	< LDL	4.54(4.28-4.91)	< LDL	NA	<0.0001^d^
Neutralization Breadth (%), median (IQR)	58.33 (25-83.33)	79.17 (50-100)	NA	NA	0.1027^e^
Geometry Median Titer (GMT), median (IQR)	19.33 (12.22-35.56)	62.10 (24.83-130.43)	NA	NA	0.0089^e^

NA, not applicable, IQR, Interquartile range. a, one-way ANOVA test; b, Chi-square test; c, Kruskal-Wallis test; d, Mann-Whitney test; e, t test.

### 3.2 Active expression of the immune and inflammatory factors in HIV-infected individuals

**Figure 1:**
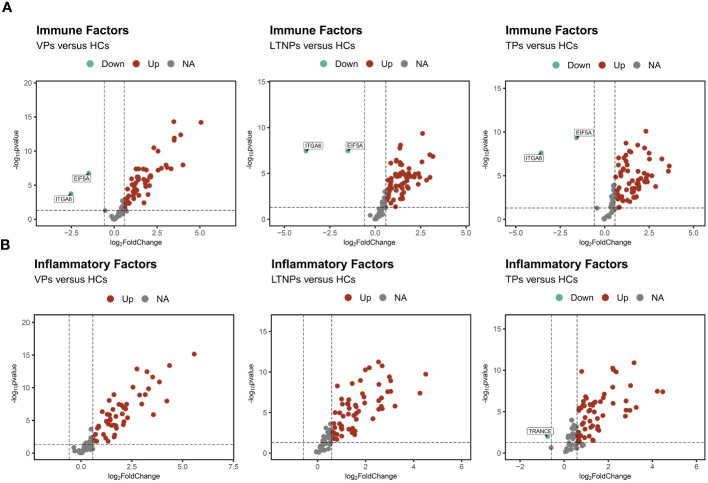
Profiles of the immune and inflammatory proteins expression levels of VPs, LTNPs and TPs. **(A)** Volcano diagram of differential protein expressions of the immune factors, with red representing up-regulated factors and green representing down-regulated factors, including LTNPs (n=13), VPs (n=11), TPs (n=16), and HCs (n=15). **(B)** Differential protein expression volcano plots of the inflammatory factors including LTNPs (n=16), VPs (n=14), TPs (n=16), and HCs (n=15). Red represents up-regulated factors, green represents down-regulated factors, and grey represents non-significant difference factors.

In comparison to HCs, we observed comparable expression profiles of the immune and inflammatory factors in VPs, LTNPs and TPs. Specifically, compared to HCs, the expression levels of immune and inflammatory factors were upregulated in the other three groups of HIV-infected individuals (**Figure 1**). Furthermore, there was a notable reduction in the expression levels of EIF5A and ITGA6 across these three groups (**Figure 1A**). Within the inflammatory factors, TRANCE displayed a significantly lower expression level in TPs compared to HCs (**Figure 1B**). Moreover, an unsupervised clustering heatmap, generated using Euclidean distance, indicated a close similarity in the expression patterns of inflammatory and immune factors between LTNPs and TPs (**SupplementaryFigure S1**).

### 3.3 DEPs characteristics between different groups

**Figure 2:**
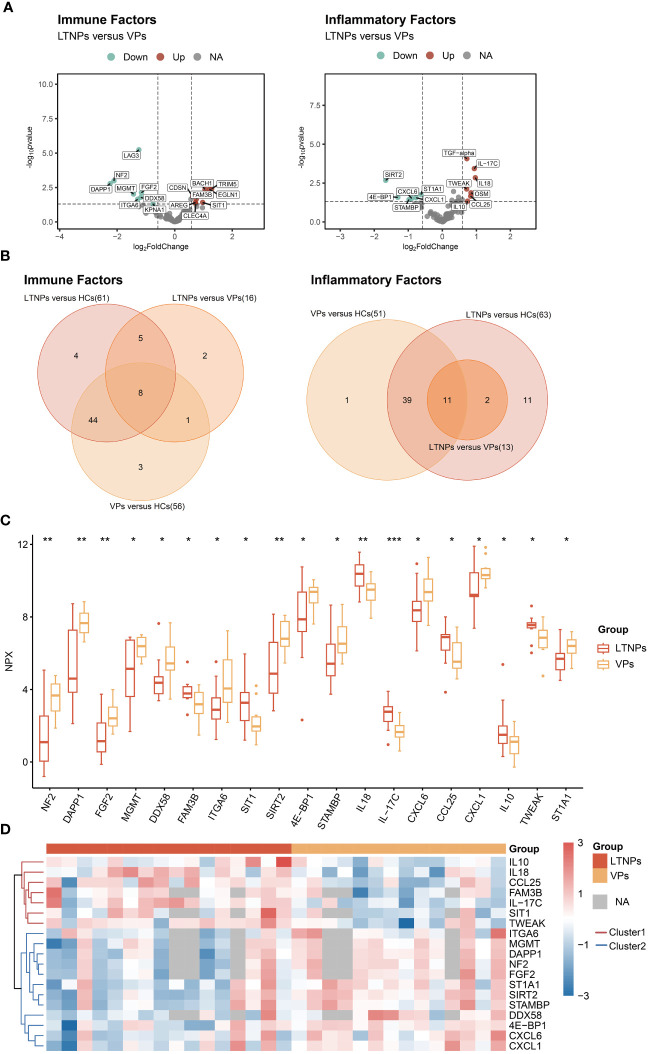
All differentially expressed immune and inflammatory factors between LTNPs and VPs. **(A)** Volcano plot depicting the expression levels of immune and inflammatory factors between LTNPs and VPs. In the left panel representing immune factors, red, green, and grey dots denote up-regulated, down-regulated, and non-significant differences, respectively, including LTNPs (n = 13) and VPs (n = 11). The right panel represents inflammation factors, including LTNPs (n = 16) and VPs (n = 14). **(B)** Venn plots of the differentially expressed immune and inflammatory factors between LTNPs and VPs. **(C)** Box plots comparing DEPs (LTNPs vs VPs) are expressed in Mean ± SD; NPX stands for Normalized Protein Expression. * p < 0.05, ** p < 0.01 and *** p < 0.001. **(D)** Heatmap of unsupervised clustering of DEPs between LTNPs and VPs. Proteins under the red or blue clustering tree are defined as Cluster1 and Cluster2, respectively.

Twenty-nine DEPs were identified between LTNPs and VPs. Among those, 16 were immune factors, and 13 were inflammatory factors (**Figure 2A**). A comparative analysis of DEPs between LTNPs, VPs and HCs showed that 19 differentially expressed proteins were shared between groups. Among the 19 proteins, eight are immune factors, and 11 are inflammation factors (**Figure 2B**). Compared to VPs, 11 out of 19 DEPs (ITGA6, MGMT, DAPP1, NF2, FGF2, STIA1, SIRT2, STAMBP, DDX58, 4E-BP1, and CXCL1) showed downregulation, whereas seven out of 19 DEPs (IL10, IL18, CCL25, FAM3B, IL-17C, SIT1, and TWEAK) exhibited upregulation in LTNPs. Based on the unsupervised clustering heatmap, we observed that these 19 DEPs could be broadly categorized into two Clusters: Cluster1 exhibited higher expression in LTNPs, while Cluster2 showed higher expression in VPs (**Figure 2D**).

Between TPs and VPs, we observed distinct expression levels (p-value <0.05) for 18 immune factors and 14 inflammatory factors (**Supplementary Figure S2A**). After further intersection analysis with HCs using the Venn diagram, we ultimately identified 19 DEPs (**Supplementary Figures S2A, B**). Specifically, in TPs, the expression levels of five DEPs (STC1, SIT1, FAM3B, TRAF2, and CCL25) were higher than those in VPs. Conversely, the expression levels of the remaining 14 DEPs (SH2D1A, MGMT, DAPP1, NF2, DDX58, CXCL9, CXCL10, CXCL11, SIRT2, CD6, IL-12B, MCP-2, ST1A1, and IL-7) were lower in TPs compared to VPs. Further investigation into the relationship between DEPs and CD4^+^ T-cell counts in TPs and VPs revealed that SIT1, FAM3B and TRAF2 had positive correlations with CD4^+^ T-cell counts, whereas SH2D1A, DAPP1, DDX58, CXCL11, CXCL9, CXCL10, IL-12B and MCP-2 presented negative correlations with CD4^+^ T-cell counts (**Supplementary Figure S2D**). The ROC analysis demonstrated that CXCL9 and CXCL10 have extremely high sensitivity in assessing the success of ART treatment. Their respective AUC values were 0.9509 and 0.9063, with p-values less than 0.0001 and 0.0002, respectively (**Supplementary Table S1**, **Supplementary Figure S3**). Interestingly, CXCL9 and CXCL10 were also the two DEPs with the strongest negative correlation with CD4^+^ T cell counts. The r values are -0.62 and -0.59 with both p values less than 0.001(**Figure S2D**).

LTNPs and TPs had similar expression profiles, and only nine inflammatory factors and one immune factor were differentially expressed between two groups (**Supplementary Figure S4A**). After further intersections with HCs by the Venn diagram, six inflammatory factors-related DEPs (TRAF2, IL-17C, CXCL9, CXCL10, CD6 and IL18) were finally identified (**Supplementary Figures S4B, C**). Five of these six DEPs (IL-17C, CXCL9, CXCL10, CD6, and IL18) were expressed at higher levels in LTNPs than in TPs, whereas TRAF2 was expressed at lower level in LTNPs than in TPs.

### 3.4 Correlation analysis of DEPs between LTNPs and VPs

**Figure 3:**
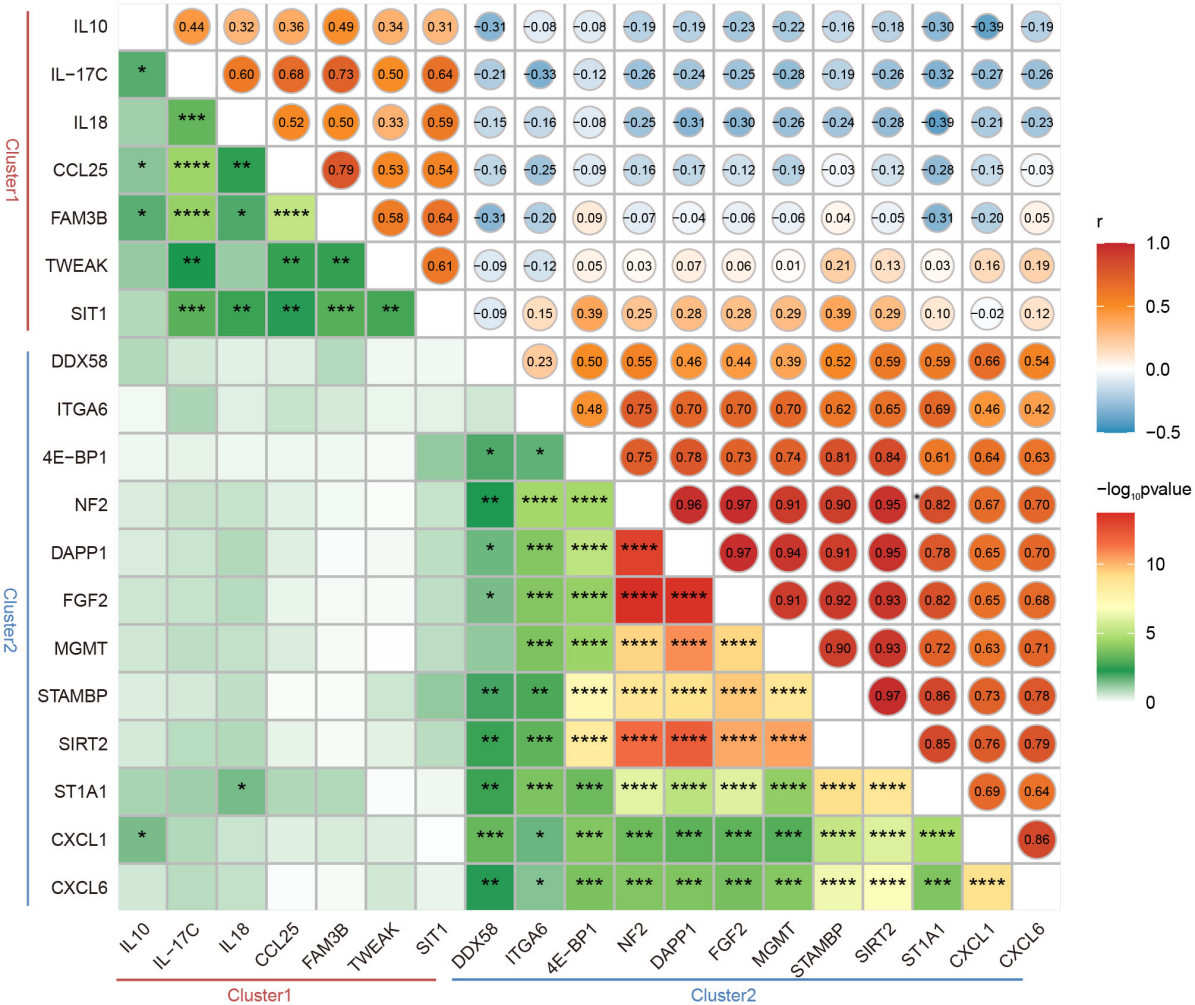
Correlation between the expression of DEPs between LTNPs and VPs. Spearman correlation matrix was used to present the correlation between the expression of DEPs. The upper triangular matrix represents correlation. The lower triangular matrix represents significance: *p <0.05, **p <0.01, ***p <0.0001 and ****p <0.0001.

The interrelations among the 19 DEPs were evaluated and categorized into two clusters according to the correlation between their expressions (**Figure 3**). Consistent with clustering in the differential protein expression heatmap (**Figure 2D**), seven factors— IL10, IL-17C, IL18, CCL25, FAM3B, TWEAK and SIT1—belong to Cluster1. Twelve factors—DDX58, ITGA6, 4E-BP1, NF2, DAPP1, FGF2, MGMT, STAMBP, SIRT2, ST1A1, CXCL1 and CXCL6—pertain to Cluster 2 (**Figure 3**). Particularly, these seven proteins in Cluster 1 were expressed at higher levels in LTNPs than in VPs, whereas those 12 proteins in Cluster 2 were expressed at lower levels in LTNPs than in VPs (**Figure 2C**).

Obviously, in Cluster 2, robust positive correlations were observed between NF2 and DAPP1, NF2 and FGF2, NF2 and MGMT, NF2 and SIRT2, NF2 and STAMBP, and NF2 and ST1A1, respectively (r-value> 0.9 and p-value < 0.0001). Furthermore, moderate negative correlations were observed between ST1A1 and IL18, CXCL1 and IL10 (r = -0.39 and p < 0.05) (**Figure 3**). These results will provide clues to the understanding of the interactions between DEPs.

### 3.5 Comprehensive analysis of DEPs between LTNPs and VPs

**Figure 4:**
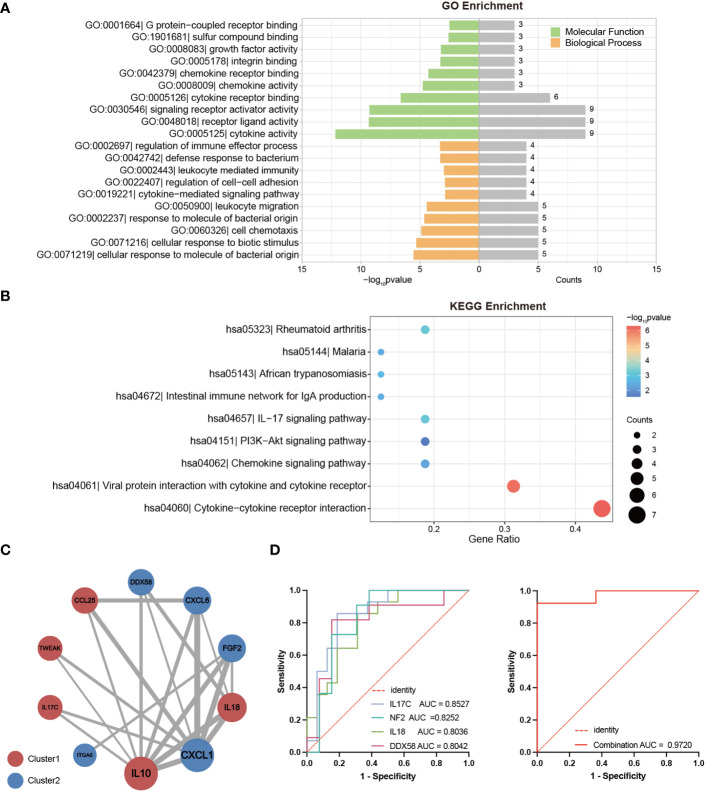
Bioinformatics Analysis of DEPs between LTNPs and VPs. **(A)** The bar graph shows the Gene Ontology (GO) enrichment analysis results based on DEPs. The left part of the enriched pathway significance -log_10_ p-value is shown by the length of the bar, where green represents Molecular Function (MF), orange represents Biological Process (BP), and the grey bar on the right represents the number of genes enriched by each pathway. **(B)** The bubble diagram is based on the results of the Kyoto Encyclopedia of Genes and Genomes (KEGG) enrichment analysis of DEPs. The bubble color represents the significance of the enriched pathway, and the bubble size represents the number of enriched genes. **(C)** The protein-protein interaction network diagram is mapped based on DEPs. The circle’s color represents a cluster: red and blue indicate Cluster1 and Cluster2, respectively. The size of the circle represents the degree. The larger the circle, the higher the degree. The thickness of the grey line represents the combined score of the interaction between the two proteins. The thicker the line, the higher the combined score. **(D)** Receiver operating characteristic curve (ROC) analysis. On the left are ROC curves for IL-17C, NF2, DDX58, and IL18 between LTNPs and VPs. On the right is the ROC curve for the combination of these four indicators between LTNPs and VPs.

To gain deeper insight into the potential functions of the DEPs between LTNPs and VPs, we conducted comprehensive analysis using Gene Ontology (GO) and Kyoto Encyclopedia of Genes and Genomes (KEGG) enrichment on the selected 19 DEPs. The GO enrichment analysis underscored the roles of these 19 DEPs are pivotal in immune response, cellular communication and intricate signaling pathways (**Figure 4A**). In detail, crucial biological processes included cellular response to molecule of bacterial origin (GO:0071219), response to molecule of bacterial origin (GO:0002237), cellular response to biotic stimulus (GO:0071216), cell chemotaxis (GO:0060326), and leukocyte migration (GO:0050900). Significant molecular functions included cytokine activity (GO:0005125), receptor ligand activity (GO:0048018), and signaling receptor activator activity (GO:0030546). Moreover, our KEGG enrichment analysis indicated that 16 of the 19 DEPs were enriched across multiple immune response and disease-related pathways (**Figure 4B**). Cytokine-cytokine receptor interaction (hsa04060) and viral protein interaction with cytokine and cytokine receptor (hsa04061) were particularly noteworthy. Remarkably, IL18, CXCL6, CXCL1, IL10 and FGF2 were consistently identified as prominently featured genes across these pathways (**Supplementary Tables S2, S3**).

Subsequently, the 19 DEPs underwent analysis using the STRING online tool for protein-protein interactions, yielding a Protein-Protein Interaction (PPI) network comprising ten proteins (**Figure 4C**). This PPI network consisted five proteins from Cluster1 (IL10, IL18, CCL25, TWEAK and IL-17C) and five proteins from Cluster2 (CXCL1, FGF2, CXCL6, DDX58 and ITGA6). Notably, IL10 and CXCL1, representatives of Cluster 1 and Cluster 2, respectively, exhibited the highest degrees (degree = 8) and may serve as key nodal proteins influencing signaling and disease control.

Then the ROC analysis results demonstrated that among the 19 DEPs, IL-17C, NF2, IL18, and DDX58 exhibited excellent diagnostic performance in distinguishing LTNP and VP, with all of their AUC values exceeding 0.8 and p-values less than 0.01 (**Supplementary Table S4**). Further analysis revealed that the combination of these four proteins significantly enhanced the sensitivity in distinguishing LTNPs and VPs, achieving an AUC of 0.9720 with p < 0.0001 (**Figure 4D**).

### 3.6 Correlation between DEPs (LTNPs vs. VPs) and clinical indicators

**Figure 5:**
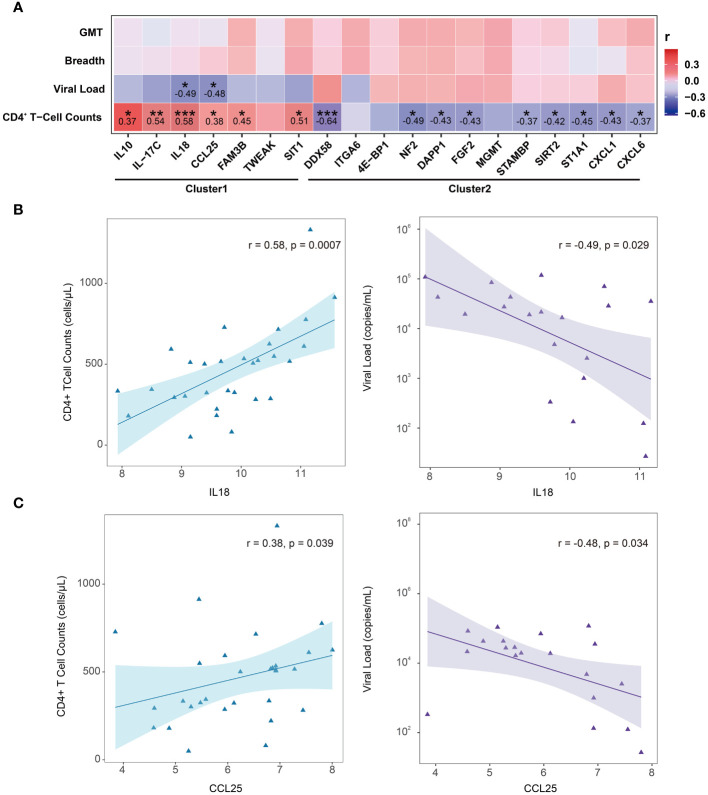
Correlation between DEPs (LTNPs vs. VPs) expression and clinical indicators. **(A)** Heatmap shows Spearman’s correlation between the expressions of DEPs (LTNPs vs. VPs) and clinical immunological indicators. * p < 0.05, ** p < 0.01 and *** p < 0.001. **(B)** The left panel shows a significant positive correlation between IL-18 and CD4^+^ T-cell counts, and the right panel shows a significant negative correlation between IL18 and viral load. **(C)** The left panel presents a significant positive correlation between CCL25 and CD4^+^ T-cell counts, and the right panel displays a significant negative correlation between CCL25 and viral load.

To further explore which DEPs (LTNPs vs. VPs) are associated with HIV disease control, a comprehensive correlation analysis of 19 DEPs with key clinical indicators, including CD4^+^ T-cell counts, viral load and plasma neutralizing activity (neutralization breadth and GMT), was performed. However, no significant correlation was observed between the 19 DEPs and plasma neutralization activity. We still found correlations between the expression levels of 15 proteins and CD4^+^ T-cell counts. The expression levels of six proteins (IL10, IL-17C, IL18, CCL25, FAM3B and SIT1) were positively correlated with CD4^+^ T-cell counts, with IL18 and IL-17C having the greatest r values of 0.58 and 0.54 (p < 0.01), respectively. The expression levels of other nine proteins (DDX58, NF2, DAPP1, FGF2, STAMBP, SIRT2, ST1A1, CXCL1 and CXCL6) were inversely correlated with the CD4^+^ T-cell counts, and DDX58 presented the lowest r value of -0.64 (p < 0.001) (**Figure 5A**). Two key node proteins, IL10 and CXCL1, identified in enrichment and PPI analysis, were positively and inversely correlated with CD4^+^ T-cell counts, respectively, with r values of 0.37 and -0.43. In addition, we found that the expression levels of IL18 and CCL25 were both positively correlated with CD4^+^ T-cell counts while also significantly negatively correlated with viral load (**Figures 5B-C**). Interestingly, the proteins whose expression levels were significantly positively correlated with CD4^+^ T-cell counts were all from Cluster1, and vice versa, from Cluster2.

### 3.7 Analysis of biomarkers that are related to plasma neutralizing activity

**Figure 6:**
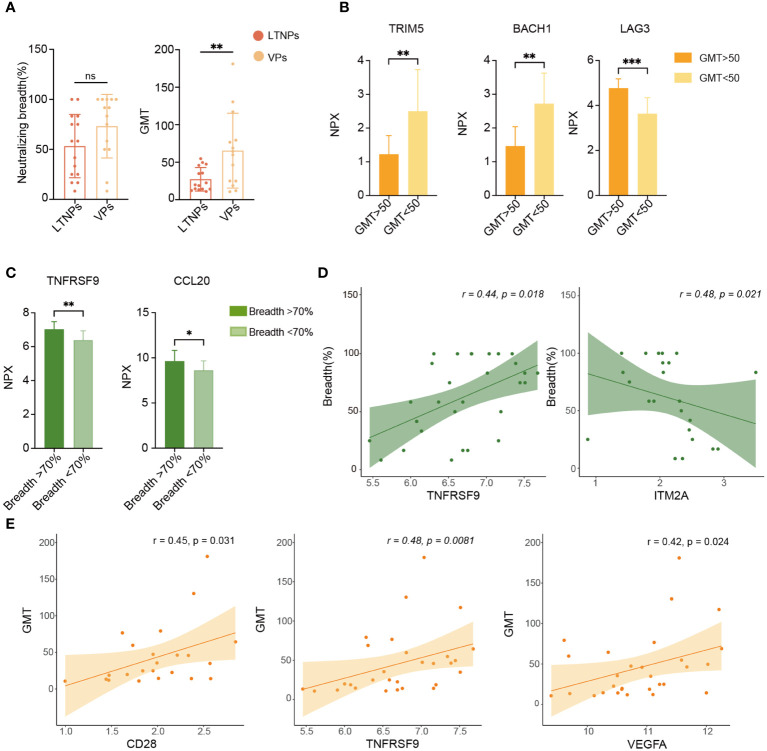
Analysis of biomarkers associated with plasma neutralizing activity. **(A)** Comparison of plasma neutralization breadth and GMT between LTNPs and VPs. **(B)** Bar plots show TRIM5, BACH1 and LAG3 expression levels. **(C)** Bar plots show TNFRSF9 and CCL20 expression levels in the two neutralization breadth groups. **(D)** Correlation of TNFRSF9 or ITM2A expression level (NPX) with neutralization breadth. **(E)** Correlation of CD28, TNFRSF9 and VEGFA expression levels (NPX) with GMT. * p < 0.05, ** p < 0.01, *** p < 0.001 and ns stands for non-significance.

To explore factors that were associated with plasma-neutralizing activity, we examined the neutralization breadth and GMT of LTNPs and VPs. Although no statistical difference was found in the neutralization breadth or GMT between LTNPs and VPs, a statistical difference in GMT was observed between the two groups (**Figure 6A**), and the GMT value in the LTNP group was significantly lower than that in the VP group (p<0.01). Then, the NPX values of all proteins were stratified using a partitioning strategy based on the criteria of GMT equal to 50 and a neutralization breadth of 70%, respectively. TRIM5 and BACH1 were down-regulated in the group of GMT>50 (p<0.001), while LAG3 levels were upregulated (p<0.0001) (**Figure 6B**), and TNFRSF9 and CCL20 expression levels were higher in the group with neutralization breadth over 70% (**Figure 6C**). Correlation analysis between plasma neutralizing activity (breadth and GMT) and each protein expression level (NPX) showed that TNFRSF9 and ITM2A were positively and negatively correlated with neutralization breadth, respectively (p<0.05), whereas CD28, TNFRSF9 and VEGFA were all positively correlated with GMT (**Figures 6D, E**).

### 3.8 Analysis of potential biomarkers between LTNPs, VPs, TPs and HCs

**Figure 7:**
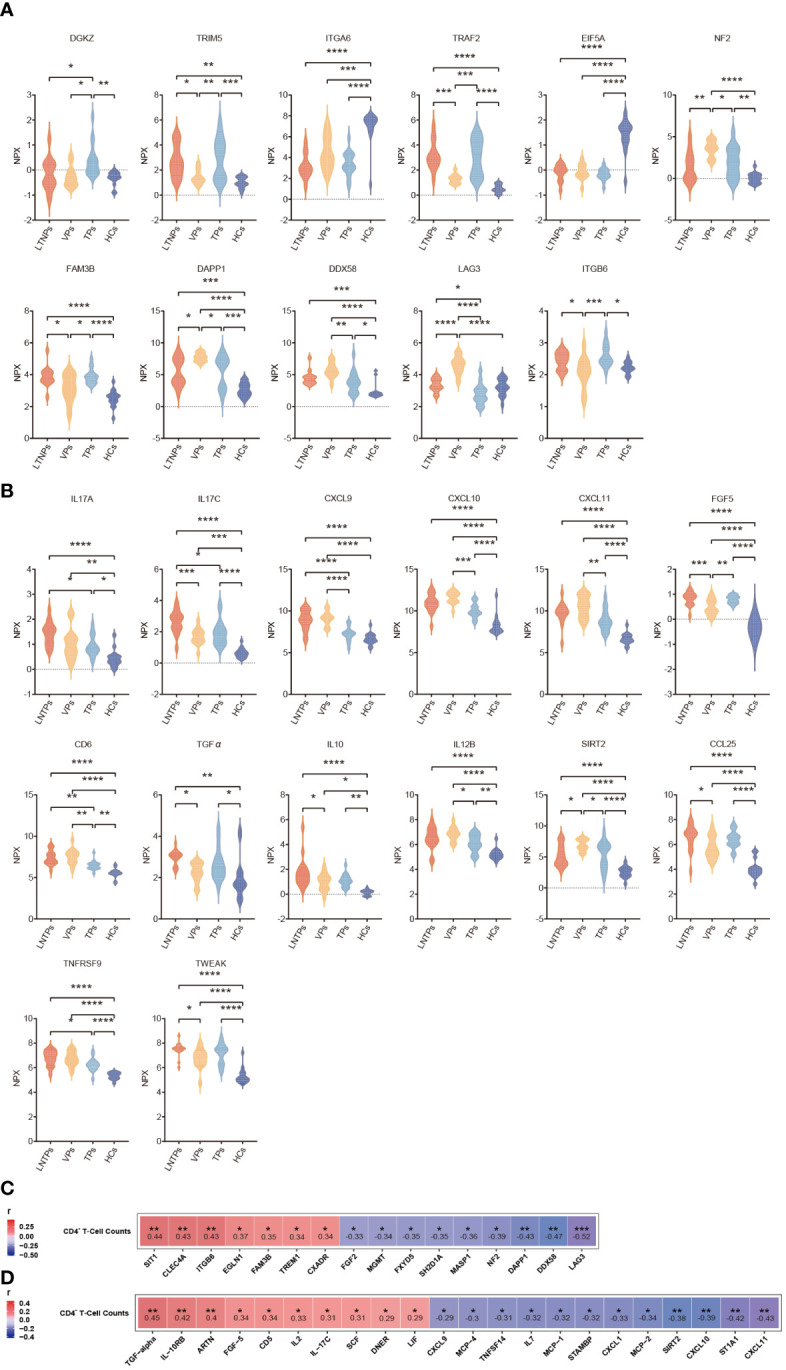
Differential analysis of the immune and inflammatory factors expression levels between the four groups. **(A)** Violin plots of the immune factors between the four groups, including LTNPs (n=13), VPs (n=11), TPs (n=16), and HCs (n=15). **(B)** Violin plots of the inflammatory factors between the four groups, including LTNPs (n=16), VPs (n=14), TPs (n=16), and HCs (n=15). **(C)** Heatmap shows the Spearman correlation between immune factors expression levels (NPX) and CD4^+^ T-cell counts. **(D)** Heatmap shows the Spearman correlation between inflammatory factors expression levels (NPX) and CD4^+^ T-cell counts. One-way ANOVA is used to evaluate the statistical difference of factors between the four groups. * p < 0.05, ** p < 0.01, *** p < 0.001 and **** p < 0.0001.

To comprehensively identify key biomarkers at different stages of HIV infection, we conducted an extensive analysis of differential protein expression levels across LTNPs, VPs, TPs and HCs. Eleven immune factors (DAPP1, DDX58, DGKZ, EIF5A, FAM3B, LAG3, NF2, TRAF2, TRIM5, ITGA6 and ITGB6) and 14 inflammatory factors (CCL25, CD6, CXCL9, CXCL10, CXCL11, FGF5, IL10, IL12B, IL-17C, IL17A, SIRT2, TGFα, TNFRSF9 and TWEAK) were found differentially expressed with statistical differences in the at least four comparisons between each group (**Figures 7A, B**). Briefly, nine DEPs were identified between LTNPs and VPs, which included four immune factors (NF2, DAPP1, DDX58 and FAM3B) and five inflammatory factors (SIRT2, IL-17C, CCL25, IL-10 and TWEAK). Moreover, a well-known immune checkpoint factor, LAG3, which is associated with immune exhaustion (24, 25), was differentially expressed in LTNPs vs. VPs, LTNPs vs. TPs, VPs vs. TPs, and VPs vs. HCs, respectively. In addition, the expression level of LAG3 in VPs was the highest among the four groups. Moreover, the expression level of LAG3 presented a significant negative correlation with CD4^+^ T-cell counts (r =-0.52 and p=0.0006) and a prominent positive correlation with viral load (r= 0.64 and p=0.0097) (**Supplementary Figures S5A-B**). This suggested that the immune system of VPs was severely challenged. In addition, TNFRSF9, which is associated with the development of follicular helper T lymphocytes (26), is expressed with higher levels in VPs and LTNPs (**Figure 7B**). However, no statistical difference of TNFRSF9 was observed between LTNPs and VPs.

Then, correlation analysis between CD4^+^ T cell-counts and expression levels of all the immune and inflammatory factors in the LTNPs, VPs and TPs revealed significant associations between special factors and CD4^+^ T cell-counts. Specifically, 16 immune factors (SIT1, CLEC4A, ITGB6, EGLN1, FAM3B, TREM1, FXADR, FGF2, MGMT, FXYD5, SH2D1A, MASP1, NF2, DAPP1, DDX58 and LAG3) and 22 inflammatory factors (TGF-α, IL-10RB, ARTN, FGF-5, CD5, IL2, IL17C, SCF, DNER, LIF, CXCL9, MCP-4, TNFSF14, IL7, MCP-1, STAMBP, CXCL1, MCP-2, SIRT2, CXCL10, ST1A1, and CXCL11) present statistically significant correlation with CD4^+^ T cell-counts (p<0. 05) (**Figures 7C, D**).

## 4 Discussion

In our study, we observed that, compared to HCs, the immune and inflammatory factors were upregulated in HIV-infected individuals, regardless of their immune status. Specifically, in TPs, the expression levels of immune and inflammatory factors were similar to those in LTNPs. In TPs and VPs, CXCL9 and CXCL10 were identified as potential auxiliary diagnostic indicators for the success of ART. Between LTNPs and VPs, we identified five proteins (IL10, CXCL1, IL18, CXCL6, and CCL25) that may associated with the immune homeostasis of LTNPs. Additionally, TNFRSF9, CD28, and VEGFA were also discovered to correlate positively with plasma neutralizing activity.

The expression levels of the immune and inflammatory factors in LTNPs, VPs and TPs were higher than those in HCs. CD4^+^ T-cell counts displayed a significant negative correlation with viral load in VPs (**Figure S5C**). The expression level of LAG3, which is an immune checkpoint associated with immune depletion, was significantly higher in VPs than in the other three groups (**Figure 6A**). As a confirmed immune checkpoint, LAG3 is mainly expressed in immune cells such as activated T cells, regulatory T cells (Treg cells) and natural killer cells (NK cells) (27). The primary function of LAG3 is to inhibit T-cell activation and function, resulting in T-cell exhaustion via binding to MHC-II (24, 25). The high expression of LAG3 in VPs was also accompanied by the low CD4^+^ T-cell count and high viral load, which mutually proved immune exhaustion and uncontrolled viral replication. Also, LAG3 deficiency in mice had been found to drive the development of effector memory CD4^+^ T-cell and enhance CD4^+^ Th-1 cell immune responses, thus reversing the trend of T cell exhaustion (25). Therefore, ART in combination with immune checkpoint inhibitors may provide a new idea for HIV treatment. However, we did not find significant differences in cytokine IL2 and IL6 between LTNPs and VPs, which is consistent with previous research findings (28). Although these factors play important roles in the progression of HIV disease, we speculate that this may be because IL2 and IL6 are markers of HIV early infection, while LTNPs and VPs in our study have lived with HIV for a longer time. Additionally, in VPs, CD4^+^T cells that secrete these factors may undergo immune exhaustion under sustained viral replication, which is in accordance with the higher expression levels of LAG3 in VPs. Unlike VPs, the expression levels of the immune and inflammatory factors in TPs receiving effective antiretroviral therapy presented the highest similarity to those in LTNPs. This also suggests that antiretroviral therapy immediately after HIV infection is effective in protecting the host’s immune function.

Five proteins were at critical nodes in the enrichment analysis of DEPs. Among them, IL10, IL18 and CCL25 belonged to Cluster1, and their expression levels were higher in LTNPs than in VPs, whereas CXCL1 and CXCL6 in Cluster2 had higher expression levels in VPs. They play roles in promoting immune function as well as inflammatory homeostasis, respectively. Despite the strong immune homeostatic regulatory function, IL-10 contributed to the establishment and persistence of viral reservoirs in SIV-infected rhesus monkeys following antiretroviral therapy (29). In another study, the high expression of IL10 and CTLA-4 in the follicular regulatory T cells (Tfr) of untreated HIV-infected patients inhibited the function of follicular helper T cells (Tfh), resulting in an imbalance between Tfr cells and Tfh cells and a deficiency in humoral immunity (30). However, in this study, we discovered that IL10 was positively correlated with CD4^+^ T-cell counts and that its level in LTNPs or VPs was higher than in VPs, indicating that IL10 had a protective effect. Thus, the role of IL10 in HIV infection needs further in-depth study.

IL18 level was positively correlated with CD4^+^ T-cell counts and inversely correlated with viral load in this research, suggesting its protective role. IL18 is reported to be an inflammasome-associated cytokine, and inflammasome in HIV cases may lead to a poorer prognosis due to excessive inflammation (31). Previous studies had shown that IL18 expression was elevated in HIV-infected patients, and its antagonist IL18-binding protein (IL18-BP) was subsequently downregulated in infected patients, and that imbalance in the dynamic balance between IL18 and IL18-BP induced viral replication in human CD4^+^ T cells (32). In another study, this imbalance between IL18 and IL18-BP was found not to occur in LTNPs, which may be the reason why normal immune function was maintained despite the higher levels of IL18 in LTNPs in our study (33).

CCL25 regulates the migration and localization of immune cells in mucosal tissues mainly by binding to CCR9 receptors, thus maintaining intestinal immune homeostasis. Furthermore, the absence of CCL25 results in the inability of CD4^+^ T expressing CCR9 and integrin α4β7 to complete homing movements to the intestinal mucosa during HIV infection (34). As observed in the study, CCL25 level was also found to be positively linked with CD4^+^ T-cell counts and negatively correlated with viral load, indicating its undirected protective effect against HIV infection.

CXCL1 and CXCL6 have a pro-inflammatory solid effect. In this research, CXCL1 and CXCL6 expression levels in VPs were much higher than those in LTNPs, indicating the active and wide inflammation status in VPs, which may be caused by uncontrolled virus propagation. Prolonged or excessive CXCL1 expression may cause the over-activation of immune cells, ultimately resulting in immune depletion (35). Similar to CXCL1, CXCL6 is also a chemokine family that regulates downstream signaling through the JAK/STAT3 pathway to exert pro-inflammatory effects (36). However, this signaling often causes inflammation-related tissue damage (37, 38). Therefore, precise regulation of CXCL1 and CXCL6 expressions is essential to avoid excessive inflammation and tissue injury.

In addition to immune modulation, we also analyzed the plasma neutralization activity of LTNPs and VPs, an important factor in containing viral infection. In this study, albeit GMT was significantly lower in LTNPs than in VPs, there was no difference in the neutralization breadth between the two groups. Further correlation analysis revealed positive correlations between GMT and TNFRSF9, GMT and VEGFA, and GMT and CD28, respectively. TNFRSF9 expression level in the group of higher neutralization breadth (>70%) was much higher than that in the lower breadth group (breadth<70%). The upregulated expression of TNFRSF9 has been reported to enhance signaling interactions with Tfh cells, which in turn enhances Tfh-B cells interactions, promotes proliferation of B-cell somatic hypermutation, and ultimately enhances antibody’s breadth and potency (26, 39). Vascular endothelial growth factor (VEGFA) belongs to the vascular endothelial growth factor family (VEGF). VEGFA usually binds to the receptor VEGFR2 for signaling and promotes vascular development (40, 41). There is evidence that ectopic expression of VEGFA promotes lymphangiogenesis, but the resulting lymphatic vessels do not function properly (42, 43). Therefore, the mechanism of VEGFA associated with neutralizing antibodies needs to be verified by further studies. CD28 serves as a secondary signaling molecule that plays a crucial role in the growth and development of Tfh cells. CD28 engages in interactions with CD80/CD86 molecules on antigen-presenting cells, thereby promoting the activation, proliferation, and differentiation of Tfh cells. This promotion contributes to the effective engagement of Tfh cells with B cells within lymphoid tissues and aids in the production of highly antigen-specific antibodies by B cells within germinal centers (44).

There are three limitations to this research. First, the sample size is small since only 16 LTNPs and 14 VPs were screened from a cohort established before 2008 using the strict criteria, and all donors received ART as part of the Chinese National Free Anti-Retroviral Treatment Program (NFAT) after 2008 and thus no longer fit this study. Second, the function of identified DEPs or biomarkers should be validated utilizing an *in vivo* or *in vitro* model. Third, we initially intended to assess the immunity differences between HIV-infected individuals at various disease stages and healthy control group. Therefore, the relatively younger healthy controls may cause slight biases between the HCs and other groups, but do not influence the comparisons between LTNPs, VPs and TPs.

## 5 Conclusion

In conclusion, the strong homeostatic regulation of the immune system allows LTNPs to suppress viral replication while also avoiding excessive inflammation that can lead to tissue damage, as well as the undesirable outcome of immune depletion. This study identified key protein markers associated with immune homeostasis during HIV infection control that played an important role in controlling disease progression or were associated with the production of neutralizing antibodies. Further studies on the regulatory pathways and mechanisms targeting these markers will contribute to the development of novel functional therapeutics as well as innovative vaccines against HIV.

## Data availability statement

The original contributions presented in the study are included in the article/**Supplementary Material**. Further inquiries can be directed to the corresponding authors.

## Ethics statement

The studies involving humans were approved by the Institutional Review Boards of the National Center for AIDS/STD Control and Prevention, Chinese Center for Disease Control and Prevention (X080216133 and X140617334). The studies were conducted in accordance with the local legislation and institutional requirements. The participants provided their written informed consent to participate in this study.

## Author contributions

WN: Data curation, Formal analysis, Methodology, Software, Visualization, Writing – original draft. LR: Methodology, Resources, Writing – review & editing. LL: Investigation, Resources, Writing – review & editing. DL: Investigation, Writing – review & editing, Funding acquisition. ZL: Writing – review & editing. MZ: Methodology, Writing – review & editing, Resources. YL: Conceptualization, Investigation, Writing – review & editing. HX: Funding acquisition, Investigation, Writing – review & editing, Resources. ZW: Conceptualization, Funding acquisition, Resources, Supervision, Validation, Writing – review & editing. YS: Conceptualization, Funding acquisition, Resources, Supervision, Writing – review & editing, Validation.
