# Supplementary material for: Plasma proteomics analysis of Chinese HIV-1 infected individuals focusing on the immune and inflammatory factors afford insight into the viral control mechanism
Source: Front Immunol. 2024 May 10;15:1378048. doi: 10.3389/fimmu.2024.1378048 (PMC11116669; doi:10.3389/fimmu.2024.1378048)
Supplement: Supplementary file 2 [file Image_1.pdf]

Supplementary figure legends

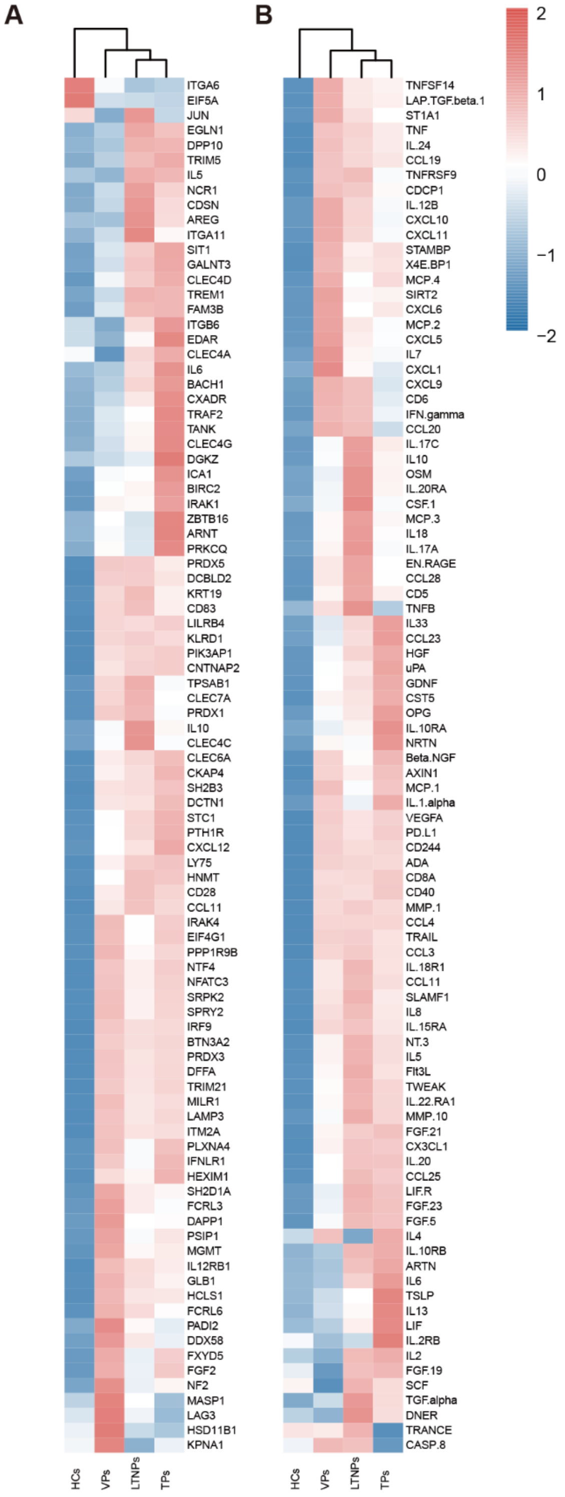

Figure S1. Heatmap of NPX in different Groups. (A) Unsupervised clustering heatmap of the expression of all immune factors measured by OLINK immune response panel, including LTNPs (n=13), VPs (n=11), TPs (n=16), and HCs (n=15). **(B)** Unsupervised clustered heatmap of all immune factor expressions measured by OLINK inflammation panel including LTNPs (n=16), VPs (n=14), TPs (n=16), and HCs (n=15).

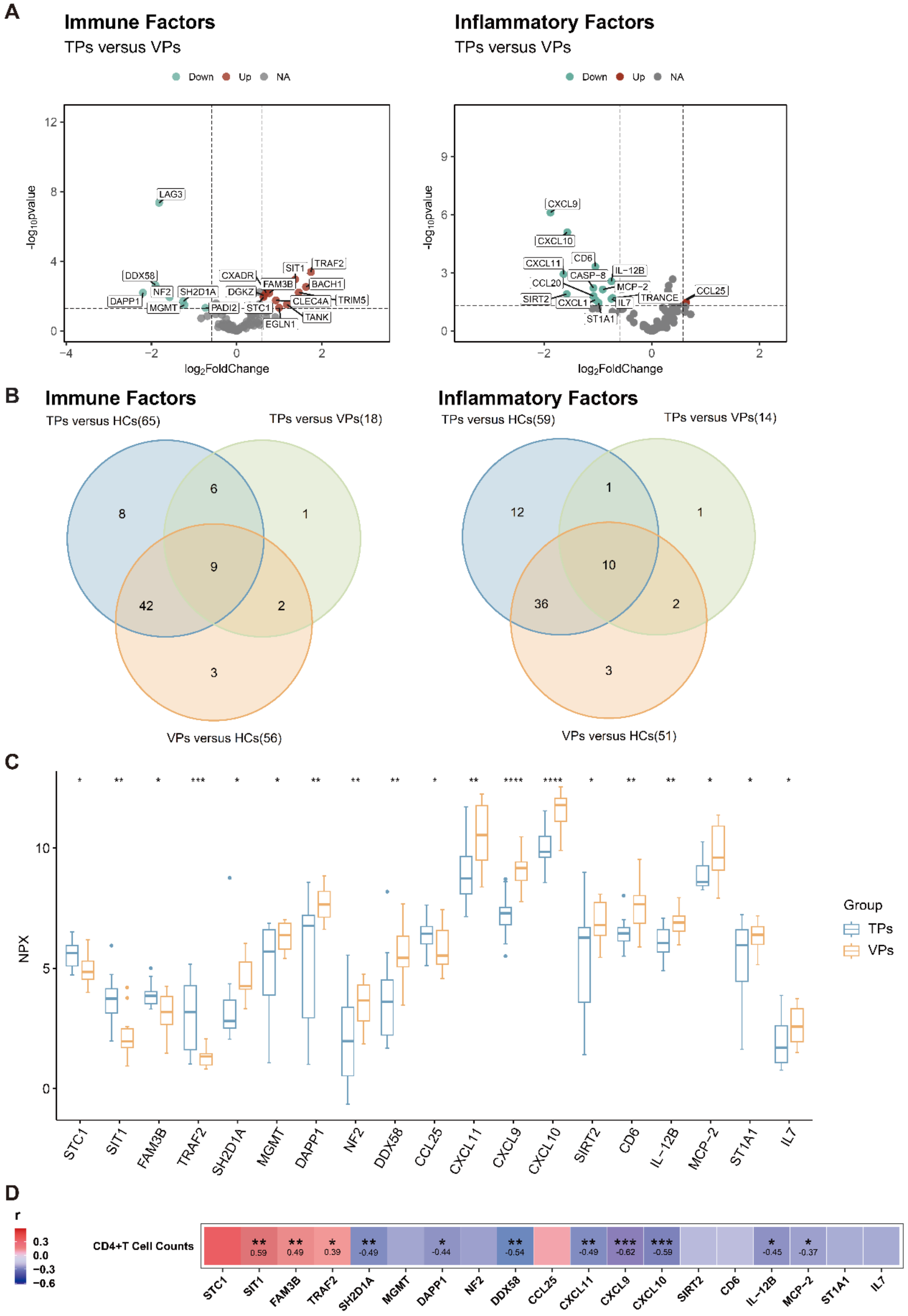

Figure S2. All differentially expressed immune and inflammation-related biomarkers between TPs and VPs. (A) Volcano plot depicting the expression levels of immune and inflammatory factors between TPs and VPs. In the left panel representing immune factors, red, green, and grey dots denote up-regulated, down-regulated, and non-

significant differences, respectively, including TPs (n = 16) and VPs (n = 11). The right panel represents inflammation factors, including TPs (n = 16) and VPs (n = 14). **(B)** Venn diagram of differential expression of the immune and inflammatory factors between TPs and VPs. The left and right panels show the immune factor Venn diagram and the inflammatory factor Venn diagram, respectively. **(C)** Box plots comparing the expression levels of DEPs (TPs vs. VPs). Data are expressed in Mean ± SD, where * $p < 0.05$, ** $p < 0.01$, *** $p < 0.001$ and **** $p < 0.0001$. **(D)** Heatmap of the correlation between the expression levels of DEPs (TPs vs. VPs) and $CD4^+$ T-cell counts. * $p < 0.05$, ** $p < 0.01$ and *** $p < 0.001$.

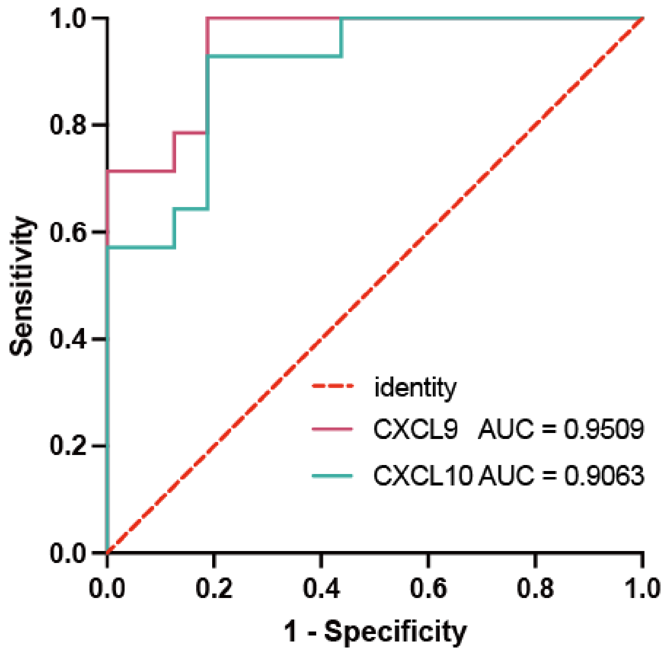

Figure S3. Receiver operating characteristic curve (ROC) analysis for CXCL9 and CXCL10 between TPs and VPs. The magenta line represents CXCL9, the cyan line represents CXCL10, and the red dashed line represents the identity, including TPs (n = 16) and VPs (n = 14).

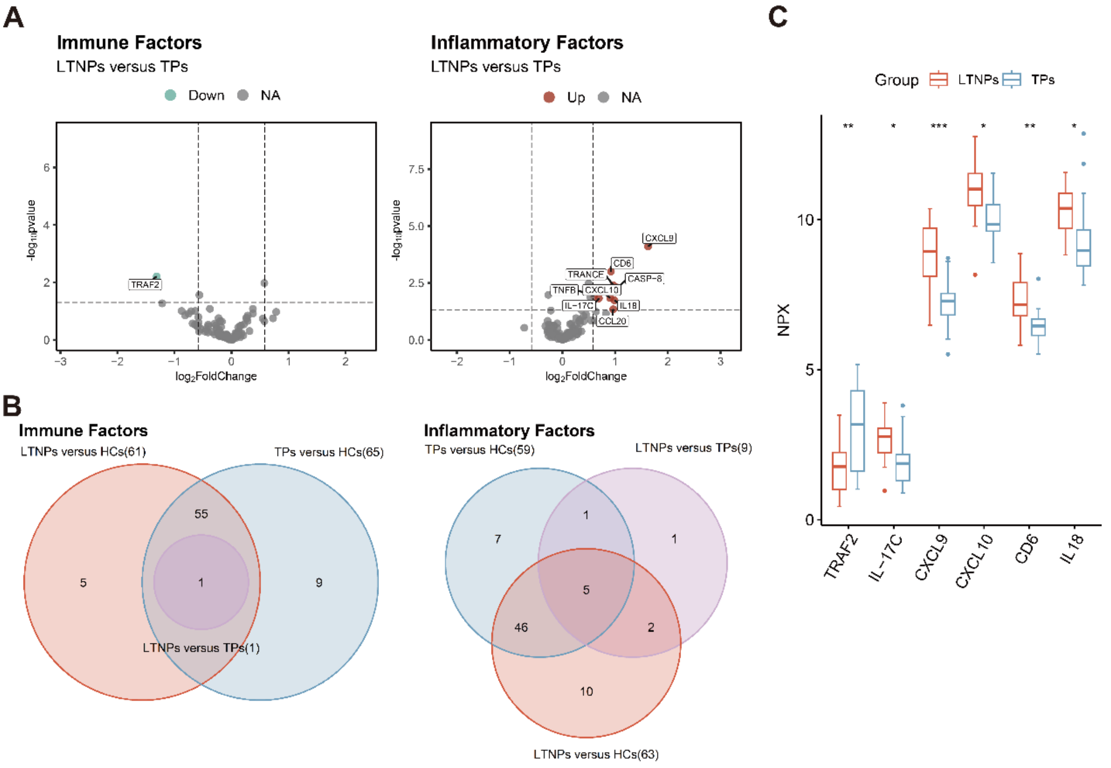

Figure S4. All differentially expressed immune and inflammation-related biomarkers between LTNPs and TPs. (A) Volcano plot depicting the expression levels of immune and inflammatory factors between TPs and VPs. In the left panel representing immune factors, red, green, and grey dots denote up-regulated, down-regulated, and non-significant differences, respectively, including LTNPs (n = 13) and TPs (n = 16). The right panel represents inflammation factors, including LTNPs (n = 16) and TPs (n = 16). **(B)** Venn plots of the immune and inflammatory DEPs between TPs and LTNPs. The left and right panels show the immune factor Venn diagrams and the inflammatory factor Venn diagrams, respectively. **(C)** DEPs box plots (TPs vs. LTNPs) are expressed in Mean ± SD, where * $p < 0.05$, ** $p < 0.01$ and *** $p < 0.001$.

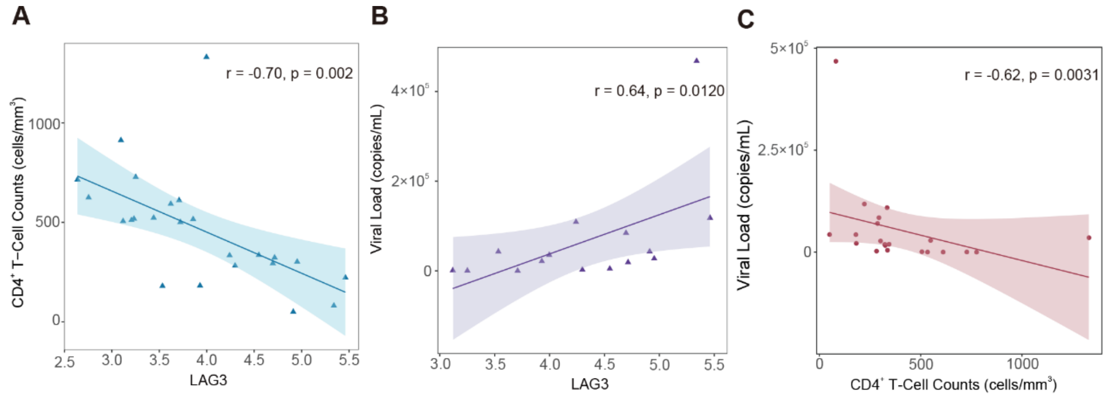

Figure S5. Correlation between NPX expression levels and clinical indicators. (A) Significant negative correlation between LAG3 and $CD4^+$ T-cell counts. **(B)** Significant positive correlation between LAG3 and Viral load. **(C)** Significant negative correlation between Viral Load and $CD4^+$ T-cell counts.
