# Supplementary material for: Plasma proteomics analysis of Chinese HIV-1 infected individuals focusing on the immune and inflammatory factors afford insight into the viral control mechanism
Source: Front Immunol. 2024 May 10;15:1378048. doi: 10.3389/fimmu.2024.1378048 (PMC11116669; doi:10.3389/fimmu.2024.1378048)
Supplement: Supplementary file 3 [file Table_1.docx]

**Supplementary Table**

**Table S1. Receiver operating characteristic curve (ROC) analysis of DEPs between TPs and VPs.**

| **ROC analysis of DEPs (TPs vs VPs)** | | | |  |
| --- | --- | --- | --- | --- |
|  |  |  |  |  |
|  | **AUC** | **95% CI** | **p-value** |  |
| **CXCL9** | **0.9509** | **0.8817 to 1.000** | **<0.0001** |  |
| **CXCL10** | **0.9063** | **0.8023 to 1.000** | **0.0002** |  |
| TRAF2 | 0.875 | 0.7450 to 1.000 | 0.0011 |  |
| SH2D1A | 0.8636 | 0.7175 to 1.000 | 0.0016 |  |
| CD6 | 0.8482 | 0.6927 to 1.000 | 0.0012 |  |
| SIT1 | 0.8409 | 0.6746 to 1.000 | 0.0031 |  |
| DDX58 | 0.8352 | 0.6706 to 0.9998 | 0.0036 |  |
| CXCL11 | 0.8214 | 0.6720 to 0.9709 | 0.0028 |  |
| IL-12B | 0.7902 | 0.6299 to 0.9505 | 0.0069 |  |
| DAPP1 | 0.7898 | 0.6191 to 0.9605 | 0.0118 |  |
| STC1 | 0.7841 | 0.5867 to 0.9815 | 0.0136 |  |
| NF2 | 0.7727 | 0.5956 to 0.9499 | 0.0179 |  |
| FAM3B | 0.7500 | 0.5412 to 0.9588 | 0.0299 |  |
| MCP-2 | 0.7411 | 0.5538 to 0.9283 | 0.0248 |  |
| IL7 | 0.7277 | 0.5436 to 0.9117 | 0.034 |  |
| SIRT2 | 0.7232 | 0.5395 to 0.9070 | 0.0377 |  |
| MGMT | 0.7216 | 0.5274 to 0.9158 | 0.0543 |  |
| CCL25 | 0.6875 | 0.4849 to 0.8901 | 0.0808 |  |
| ST1A1 | 0.6563 | 0.4551 to 0.8574 | 0.1457 |  |

**Table S2. GO enrichment analysis of DEPs between LTNPs and VPs.**

|  | Description | GeneRatio | -log10pvalue | geneID | Counts |
| --- | --- | --- | --- | --- | --- |
| BP | **GO:0071219\| cellular response to molecule of bacterial origin** | 5/19 | 5.55112859 | SIRT2/IL18/CXCL6/CXCL1/IL10 | 5 |
| BP | **GO:0071216\| cellular response to biotic stimulus** | 5/19 | 5.319351036 | SIRT2/IL18/CXCL6/CXCL1/IL10 | 5 |
| BP | **GO:0060326\| cell chemotaxis** | 5/19 | 4.896219214 | FGF2/CXCL6/CCL25/CXCL1/IL10 | 5 |
| BP | **GO:0002237\| response to molecule of bacterial origin** | 5/19 | 4.643192016 | SIRT2/IL18/CXCL6/CXCL1/IL10 | 5 |
| BP | **GO:0050900\| leukocyte migration** | 5/19 | 4.434423296 | ITGA6/CXCL6/CCL25/CXCL1/IL10 | 5 |
| BP | **GO:0019221\| cytokine-mediated signaling pathway** | 4/19 | 2.877211019 | IL18/CXCL6/CCL25/CXCL1 | 4 |
| BP | **GO:0022407\| regulation of cell-cell adhesion** | 4/19 | 2.896749914 | NF2/IL18/CCL25/IL10 | 4 |
| BP | **GO:0002443\| leukocyte mediated immunity** | 4/19 | 2.988017145 | DDX58/IL18/CXCL6/IL10 | 4 |
| BP | **GO:0042742\| defense response to bacterium** | 4/19 | 3.300791394 | SIRT2/IL18/CXCL6/IL10 | 4 |
| BP | **GO:0002697\| regulation of immune effector process** | 4/19 | 3.313709663 | RIGI/IL18/CXCL6/IL10 | 4 |
| MF | **GO:0005125\| cytokine activity** | 9/19 | 12.16640186 | FGF2/FAM3B/IL18/IL17C/CXCL6/CCL25/CXCL1/IL10/ TWEAK | 9 |
| MF | **GO:0048018\| receptor ligand activity** | 9/19 | 9.339421768 | FGF2/FAM3B/IL18/IL17C/CXCL6/CCL25/CXCL1/IL10/ TWEAK | 9 |
| MF | **GO:0030546\| signaling receptor activator activity** | 9/19 | 9.285156393 | FGF2/FAM3B/IL18/IL17C/CXCL6/CCL25/CXCL1/IL10/ TWEAK | 9 |
| MF | **GO:0005126\| cytokine receptor binding** | 6/19 | 6.637117538 | IL18/CXCL6/CCL25/CXCL1/IL10/TWEAK | 6 |
| MF | **GO:0008009\| chemokine activity** | 3/19 | 4.779800252 | CXCL6/CCL25/CXCL1 | 3 |
| MF | **GO:0042379\| chemokine receptor binding** | 3/19 | 4.29435322 | CXCL6/CCL25/CXCL1 | 3 |
| MF | **GO:0005178\| integrin binding** | 3/19 | 3.282624745 | NF2/FGF2/ITGA6 | 3 |
| MF | **GO:0008083\| growth factor activity** | 3/19 | 3.234837927 | FGF2/CXCL1/IL10 | 3 |
| MF | **GO:1901681\| sulfur compound binding** | 3/19 | 2.596590508 | FGF2/CXCL6/ST1A1 | 3 |
| MF | **GO:0001664\| G protein-coupled receptor binding** | 3/19 | 2.513041344 | CXCL6/CCL25/CXCL1 | 3 |

**Table S3. KEGG enrichment analysis of DEPs between LTNPs and VPs.**

| Description | GeneRatio | -log10pvalue | geneID | Counts | |
| --- | --- | --- | --- | --- | --- |
| hsa05143\|African trypanosomiasis | 2/16 | 1.459366233 | IL18/IL10 | | 2 |
| hsa04672\|Intestinal immune network for IgA production | 2/16 | 1.346968212 | CCL25/IL10 | | 2 |
| hsa05144\|Malaria | 2/16 | 1.346968212 | IL18/IL10 | | 2 |
| hsa05134\|Legionellosis | 2/16 | 1.337267533 | IL18/CXCL1 | | 2 |
| hsa05323\|Rheumatoid arthritis | 3/16 | 1.87424952 | IL18/CXCL6/CXCL1 | | 3 |
| hsa04657\|IL-17 signaling pathway | 3/16 | 1.87424952 | IL17C/CXCL6/CXCL1 | | 3 |
| hsa04062\|Chemokine signaling pathway | 3/16 | 1.337267533 | CXCL6/CCL25/CXCL1 | | 3 |
| hsa04061\|Viral protein interaction with cytokine and cytokine receptor | 5/16 | 4.496223333 | IL18/CXCL6/CCL25/CXCL1/IL10 | | 5 |
| hsa04060\|Cytokine-cytokine receptor interaction | 7/16 | 4.496223333 | IL18/IL17C/CXCL6/CCL25/CXCL1/IL10/TWEAK | | 7 |

**Table S4. Receiver operating characteristic curve (ROC) analysis of DEPs between LTNPs and VPs.**

| **ROC analysis of DEPs (LTNPs vs VPs)** | | | |
| --- | --- | --- | --- |
|  |  |  |  |
|  | **AUC** | **95% CI** | **p-value** |
| **IL17C** | **0.8527** | **0.7108 to 0.9945** | **0.001** |
| **NF2** | **0.8252** | **0.6479 to 1.000** | **0.0071** |
| **DDX58** | **0.8042** | **0.6127 to 0.9957** | **0.0117** |
| **IL18** | **0.8036** | **0.6472 to 0.9599** | **0.0047** |
| **Combination of IL-17C，NF2，DDX58 and IL8** | **0.9720** | **0.9112 to 1.000** | **<0.0001** |
| DAPP1 | 0.7972 | 0.6139 to 0.9805 | 0.0138 |
| TWEAK | 0.7902 | 0.6034 to 0.9770 | 0.0069 |
| FGF2 | 0.7902 | 0.6029 to 0.9775 | 0.0162 |
| CCL25 | 0.7723 | 0.5984 to 0.9463 | 0.0112 |
| SIRT2 | 0.7723 | 0.5926 to 0.9521 | 0.0112 |
| MGMT | 0.7692 | 0.5746 to 0.9639 | 0.0257 |
| ST1A1 | 0.7545 | 0.5689 to 0.9401 | 0.0178 |
| FAM3B | 0.7483 | 0.5423 to 0.9542 | 0.0397 |
| SIT1 | 0.7343 | 0.5251 to 0.9435 | 0.0523 |
| ITGA6 | 0.7343 | 0.5267 to 0.9419 | 0.0523 |
| STAMBP | 0.7321 | 0.5467 to 0.9176 | 0.0306 |
| CXCL6 | 0.7232 | 0.5337 to 0.9127 | 0.0377 |
| 4E-BP1 | 0.7188 | 0.5310 to 0.9065 | 0.0417 |
| CXCL1 | 0.7143 | 0.5164 to 0.9122 | 0.046 |
| IL10 | 0.6964 | 0.5067 to 0.8861 | 0.0674 |
